# CEO Dimensions and Their Impact on Innovation Capabilities: An Empirical Study of Colombian MSMEs

**DOI:** 10.12688/f1000research.171456.1

**Published:** 2025-11-26

**Authors:** Tobias Alfonso Parodi-Camano, Jhon Víctor Vidal Durango, Iván Portnoy

**Affiliations:** 1 Industrial Engineering Department, Universidad de Cordoba, Montería, Cordoba, Colombia; 2 National Directorate of Research, Corporacion Unificada Nacional de Educacion Superior, Bogotá, Bogota, Colombia; 3 Innovation and Productivity Department, Universidad de la Costa, Barranquilla, Atlantico, Colombia; 4 Faculty of Basic Sciences, Universidad de Cordoba, Montería, Cordoba, Colombia

**Keywords:** CEO personality, HEXACO model, transformational leadership, innovative behavior, innovation capabilities, MSMEs

## Abstract

**Background:**

Innovation in micro, small, and medium-sized enterprises (MSMEs) often stems from CEO traits and leadership. This study examines how CEO personality, transformational leadership, and innovative behavior drive innovation capabilities in Colombian MSMEs.

**Methods:**

Grounded in the HEXACO model and transformational leadership theory, a mediation model was tested using data from 106 CEOs. Structural equation modeling (SEM) assessed relationships among Openness to Experience, Conscientiousness, leadership, innovative behavior, and innovation capabilities.

**Results:**

The model showed good fit (CFI = 0.92, TLI = 0.90, RMSEA = 0.09, SRMR = 0.07). Openness and Conscientiousness significantly predicted transformational leadership, which in turn promoted innovative behavior. Innovative behavior enhanced firms’ innovation capabilities, confirming mediation from CEO traits through leadership to organizational outcomes.

**Conclusions:**

CEO personality and leadership are key to fostering innovation in resource-constrained MSMEs. The findings extend upper-echelons theory and suggest leadership development is key to improving innovation performance in emerging-economy MSMEs.

## 1. Introduction

Innovation is not a spontaneous process but rather the result of deliberate decisions made by leaders who shape their organizations. Behind every strategic choice that drives a company forward, there is a leader—often the CEO—whose vision, personality, and leadership style determine how opportunities are recognized and resources are mobilized. This influence becomes even more crucial in micro, small, and medium-sized enterprises (MSMEs), where structural limitations require agility, creativity, and a strong sense of direction. In such environments, the CEO plays a role not just as a decision-maker, but as a catalyst for organizational learning, experimentation, and transformation.

The Upper Echelons Theory ( Hambrick & Mason, 1984) offers a compelling approach to understanding this phenomenon, suggesting that organizational outcomes reflect the values and cognitive bases of top executives. Over the past decade, scholars have increasingly explored how CEO traits influence firm-level innovation. Attributes such as openness to experience, conscientiousness, and emotional stability have been associated with proactive innovation behaviors ( Zettler, Thielmann, Hilbig, & Moshagen, 2020), while narcissism has revealed a dual nature—driving ambition and risk-taking, but at times compromising long-term value ( Parodi-Camano, Vidal-Durango, & Portnoy, 2025; Zhu, Jia, Zhang, & Wang, 2025). Beyond individual traits, leadership style also matters. Transformational leaders who inspire, challenge, and support their teams often foster cultures where innovation thrives ( Bass & Riggio, 2006; Jun & Lee, 2023).

However, much of the existing literature focuses on large corporations in developed economies, overlooking the complex realities of MSMEs in emerging markets. Additionally, few studies have integrated multidimensional personality models—such as the HEXACO framework—with leadership theory to explore their combined effects on innovation. HEXACO traits such as openness to experience and conscientiousness have been shown to influence both leadership behaviors and organizational outcomes, yet their role in innovation within MSMEs remains underexplored ( Ashton & Lee, 2009; O’Reilly, Cao, & Sull, 2025). Furthermore, CEO leadership styles—particularly transformational leadership—have been identified as key factors in driving organizational change and innovation, yet the mechanisms through which these traits interact with innovative behavior are still unclear ( Ling, Simsek, Lubatkin, & Veiga, 2008; Pan, Verbeke, & Yuan, 2021). Even fewer studies have addressed these issues using comprehensive, validated instruments capable of capturing both dispositional and behavioral predictors of innovation capabilities. This study addresses these gaps by examining the combined effects of CEO traits, leadership style, and innovative behavior on innovation capabilities in MSMEs in Colombia.

This research addresses these gaps by focusing on MSMEs in Colombia, a country whose innovation potential remains largely untapped due to persistent structural barriers. Limited investment in research and development (R&D), weak linkages between academia and industry, and technological dependency continue to constrain innovation performance ( Restrepo-Morales, Loaiza, & Vanegas, 2019; Tegethoff, Santa, Bucheli, Cabrera, & Scavarda, 2025). Within this landscape, the role of the CEO is amplified. Their ability to lead, adapt, and mobilize resources becomes critical to overcoming external limitations. MSMEs—representing over 90% of the country’s business sector—are both vulnerable to these constraints and vital to economic development ( CEPAL, 2022).

By examining Colombian MSMEs across a range of industrial sectors, this study seeks to uncover how CEO personality traits, leadership style, and innovation-oriented behaviors interact to shape firms’ innovation capabilities. Drawing on three validated instruments—the HEXACO Personality Inventory ( Ashton & Lee, 2008a, 2008b; Lee & Ashton, 2008), a six-dimensional model measuring traits such as Honesty-Humility, Emotionality, and Openness to Experience; the Multifactor Leadership Questionnaire ( Bass & Avolio, 1996; Batista-Foguet, Esteve, & van Witteloostuijn, 2021), which evaluates transformational, transactional, and laissez-faire leadership styles; and an Innovation Behavior Scale ( Salessi & Etchevers, 2020), which assesses the generation, promotion, and realization of innovative ideas—this study contributes empirical evidence to a growing but still fragmented field. In doing so, it offers theoretical and practical insights for those interested in leadership, innovation, and the development of resilient innovation ecosystems in emerging economies.

## 2. Methods

### 2.1 Study design

This study employed a cross-sectional, correlational research design aimed at identifying relationships between CEO dimensions—specifically personality traits, leadership style, and innovation behavior—and the innovation capabilities of MSMEs. The model is grounded in the Upper Echelons Theory and integrates constructs from the HEXACO personality framework, transformational leadership theory, and behavioral innovation literature. Data were collected through validated psychometric instruments and analyzed using structural equation modeling (SEM) ( Hair Jr et al., 2021).

This design allows for an understanding of CEO attributes over time without establishing causality and emphasizes the cross-sectional nature of the data collection, which was conducted at a single point in time. As noted by ( Darouichi, Kunisch, Menz, & Cannella Jr, 2021), CEO tenure is a critical factor that influences strategic decision-making and performance, affecting both organizational behavior and long-term innovation capabilities. While CEO tenure has often been viewed as a determinant of leadership effectiveness, it remains an underexplored construct in the MSMEs context, especially in emerging markets like Colombia. This research fills that gap by examining how CEO tenure interacts with personality traits and leadership style, ultimately affecting innovation outcomes in firms under resource constraints.

### 2.2 Participants and sampling

The target population consisted of CEOs or top executives of MSMEs located in Colombia. A non-probability, purposive sampling method was employed, considering accessibility and the inclusion criteria of being the top decision-maker within the firm. Given the limited access to high-level executives in MSMEs, a convenience sampling approach was deemed acceptable and practical for this study. A total of 106 valid responses were obtained. The sample covered firms from diverse economic sectors, including services, manufacturing, agribusiness, and technology.

### 2.3 Instruments

**Personality traits:**

CEO personality was assessed using the HEXACO-60 inventory ( Ashton & Lee, 2009), a validated Spanish-language adaptation that measures six dimensions: Honesty-Humility, Emotionality, Extraversion, Agreeableness, Conscientiousness, and Openness to Experience. The inventory consists of 10 items per dimension, totaling 60 items with a 5-level Likert scale.

**Leadership style:**

The Multifactor Leadership Questionnaire (MLQ-5X Short), adapted for the Colombian context, was used to evaluate transformational leadership. This instrument includes 10 items (also with a 5-point Likert scale) assessing the following dimensions: transformational leadership, transactional leadership, and passive leadership ( Bass & Riggio, 2006). The internal consistency for the MLQ subscales has consistently shown Cronbach’s alpha values greater than 0.80.

**Innovative behavior:**

CEO innovation behavior was measured using a custom scale adapted from the work of Janssen ( Janssen, 2000) and validated by Bedoya et al. ( Antonio Bedoya, Pérez, Zapata-Molina, Baier-Fuentes, & Yenny Hernández, 2024; Bedoya, Pérez-Sanchéz, Zapata, Baier-Fuentes, & Hernandez-Sanchez, 2024), which captures the following dimensions: questioning, exploration-experimentation, achievement motivation, need for conformity, observation, idea networks, and tolerance for ambiguity. The scale includes 10 items, with a 5-point Likert scale used to assess CEO responses. Items assess both the frequency and quality of innovative behaviors demonstrated by CEOs.

**Innovation capabilities:**

Organizational innovation capabilities were assessed using a structured questionnaire, proposed by Calik et al. ( Calik, Calisir, & Cetinguc, 2017), that incorporated five core dimensions: creation of new products and services, enhancement of internal processes, marketing innovation, knowledge management, and investment in research and development. This measurement approach draws on the dynamic capabilities framework developed by Teece ( Teece, 2007) and has been adopted in prior research to evaluate firms’ capacity to adapt and generate value in dynamic environments ( Edeh et al., 2022).

All instruments demonstrated satisfactory psychometric properties, including internal reliability and construct validity, based on prior applications and pilot testing conducted during this study.

### 2.4 Data collection

Data were gathered through a self-administered online questionnaire using Google Forms. Participation was voluntary and anonymous. Informed consent was obtained from all participants before they began the questionnaire, ensuring they understood the study’s purpose and their rights as participants. Prior to full deployment, the instrument was piloted with a subsample of 106 CEOs to assess clarity and response time. Minor adjustments were made based on participant feedback. Data collection took place between May and July 2025.

### 2.5 Statistical analysis

Descriptive statistics were calculated to summarize sample characteristics and variable distributions. Then, we calculated the Pearson correlation coefficients for all pairs of dimensions across the constructs. The statistical significance of these correlations was then evaluated using Fisher’s Z test at the 95% confidence level ( Obilor & Amadi, 2018).

Reliability analyses to assess the data’s factorability were conducted on all scales using the Bartlett’s test of sphericity ( Bartlett, 1951), the Kaiser-Meyer-Olkin (KMO) test ( Dziuban & Shirkey, 1974; Kaiser, 1974), and the Cronbach’s alpha values ( Tavakol & Dennick, 2011). To test the proposed model, SEM using maximum likelihood estimation was applied to examine the hypothesized relationships between CEO dimensions and innovation capabilities. Model fit was assessed through standard indices: the comparative fit index (CFI), Tucker Lewis index (TLI), root-mean-squared error of approximation (RMSEA), and standardized root mean square residual (SRMR) ( Rosseel, 2012).

All analyses were conducted using R (version 4.1.2) for statistical tests and structural equation modeling. Descriptive statistics, correlation analysis, and confirmatory path analysis were performed to test the hypothesized relationships among CEO traits, leadership style, innovative behavior, and innovation capabilities. The lavaan package in R ( Rosseel, 2012) was used to perform SEM, while other statistical tests (e.g., Pearson correlation) were conducted using base R functions. The source code and the datasets are freely available at https://github.com/iportnoy1/CEO-Dimensions-and-Their-Impact-on-Innovation-Capabilities-An-Empirical-Study-of-Colombian-MSMEs - and https://zenodo.org/records/ 17609131 under an MIT and a CC BY 4.0 License, respectively.

### 2.6 Ethical considerations

No ethics committee approval was required for this study, as it involved voluntary participation and the collection of anonymous, de-identified data. Informed consent was obtained electronically from all participants prior to data submission, ensuring that they were fully informed about the purpose of the study and their rights.

### 2.7 Theoretical model and hypotheses

This study proposes a theoretical model that explores how CEO personality traits, leadership style, and innovative behavior contribute to the development of innovation capabilities in MSMEs. The model integrates four conceptual domains: (1) the HEXACO personality framework, (2) transformational leadership theory, (3) the behavioral dimensions of innovative work behavior, and (4) the innovation capabilities dimensions.

Based on the reviewed literature, the following hypotheses are formulated:

• **H1:** CEO personality traits related to Openness to Experience and Conscientiousness (as defined by the HEXACO model) are positively associated with transformational leadership style ( Zettler et al., 2020).
• **H2:** Transformational leadership is positively associated with innovative behavior among CEOs ( Bass & Riggio, 2006; Kim & Yoon, 2025).
• **H3:** Innovative behavior is positively associated with the firm’s innovation capabilities ( Hock-Doepgen, Montasser, Klein, Clauss, & Maalaoui, 2025; Janssen, 2000).
• **H4:** Transformational leadership mediates the relationship between CEO personality traits and innovative behavior ( Yu & Xiang, 2024).
• **H5:** CEO personality traits have an indirect effect on innovation capabilities through the sequential mediation of transformational leadership and innovative behavior ( Kim & Yoon, 2025).

**Figure 1.**
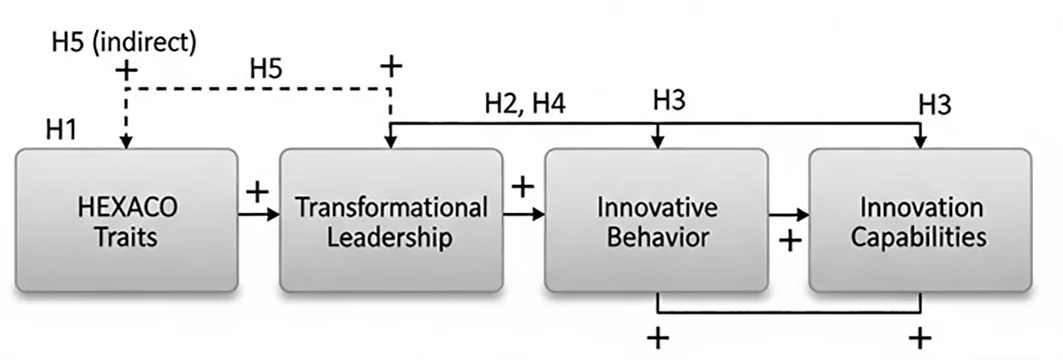
Theoretical model of the influence of CEO dimensions on innovation capabilities.

The relationships hypothesized in this study are visually represented in Figure 1, which illustrates the proposed model linking CEO personality traits, transformational leadership, and innovative behavior with innovation capabilities in MSMEs.

We tested our hypotheses using SEM with the lavaan package in R ( Rosseel, 2012). The model was theory-driven, reflecting hypothesized links between CEO personality traits (HEXACO dimensions), transformational leadership, innovative behavior, and innovation capabilities. Specifically, Openness to Experience and Conscientiousness were modeled as exogenous predictors of transformational leadership, which in turn was expected to influence innovative behavior and, subsequently, innovation capabilities.

To ensure measurement validity, we modeled Innovative Behavior (seven subscales) and Innovation Capabilities (six subscales) as latent constructs, while Transformational Leadership was included as a manifest composite variable derived from its corresponding items. This approach balances parsimony and theoretical clarity while maintaining adequate reliability.

## 3. Results

### 3.1 Descriptive statistics

An exploratory analysis was conducted using responses from **106 CEOs** representing MSMEs in various Colombian regions. Table 1 presents the means, standard deviations, and range (minimum and maximum values) of the key constructs measured: CEO personality traits (HEXACO model), transformational leadership, innovative behavior, and perceived innovation capabilities.

**Table 1.** Descriptive statistics of main constructs (n = 106).

Construct	Mean	St. Dev.	Min	Max
HEXACO Traits	3.04	0.36	2.19	4.39
Transformational Leadership	3.95	0.34	3.02	4.69
Innovative Behavior	4.04	0.49	2.79	5.00
Innovation Capabilities	3.88	0.41	2.95	4.73

The results show moderate to high mean scores across all dimensions. HEXACO traits averaged 3.04 (SD = 0.36), suggesting a generally high self-assessment of key personality attributes. Transformational leadership reported a mean of 3.95 (SD = 0.34), while innovative behavior exhibited slightly higher values (M = 4.04, SD = 0.49), indicating active involvement in innovation-related practices. Innovation capabilities also scored relatively high (M = 3.88, SD = 0.41), reflecting a positive perception among CEOs regarding their firms’ ability to innovate. Table 2 shows the factorability assessment metrics.

**Table 2.** Factorability analysis outcomes.

Questionnaire	Dimension	Cronbach’s Alpha	Mean	SD	KMO	Bartlett’s p-value
HEXACO	Honesty-Humility	0.62	3.64	0.5	0.6	2.05E-99
	Emotionality	0.62	3.24	0.32		
	Extraversion	0.61	3.5	0.57		
	Agreeableness	0.55	3.32	0.47		
	Conscientiousness	0.79	3.83	0.62		
	Openness to Experience	0.67	3.59	0.62		
Leadership	Transformational Leadership	0.87	4.27	0.43	0.83	8.67E-156
	Transactional Leadership	0.68	3.5	0.32		
	Passive Leadership	0.72	3.49	0.56		
Innovative Behavior	Questioning	0.69	3.76	0.42	0.85	2.56E-128
	Exploration-Experimentation	0.76	3.85	0.73		
	Achievement Motivation	0.83	4.42	0.57		
	Need for Conformity	0.70	2.63	0.5		
	Observation	0.77	4.27	0.65		
	Idea Networks	0.75	3.78	0.83		
	Tolerance for Ambiguity	0.56	3.49	0.49		
Innovation Capabilities	Product Innovation	0.76	4.01	0.75	0.93	1.21E-283
	Process Innovation	0.60	3.81	0.79		
	Organizational Innovation	0.79	3.6	0.94		
	Marketing Innovation	0.83	3.88	0.86		
	Innovation Culture	0.84	3.74	0.88		
	Resources for Innovation	0.94	3.67	0.89		

From Table 2, it is noticeable that Cronbach’s alpha values indicate that Leadership, Innovation Capabilities, most Innovative Behavior subscales, and most HEXACO subscales achieved acceptable reliability. In contrast, some HEXACO dimensions and the Tolerance for Ambiguity (Innovative Behavior) subscale displayed low reliability, warranting cautious interpretation if these dimensions are involved in the main hypotheses.

These findings provide an initial overview of the psychological and behavioral attributes of the CEOs in the sample and set the foundation for examining correlations and causal paths in subsequent analyses.

### 3.2 Correlational analysis

To assess the relationships among the key constructs, Pearson correlation coefficients were computed. The analysis examined the associations between CEO personality traits (HEXACO dimensions), transformational leadership, innovative behavior, and innovation capabilities dimensions.

**Figure 2.**
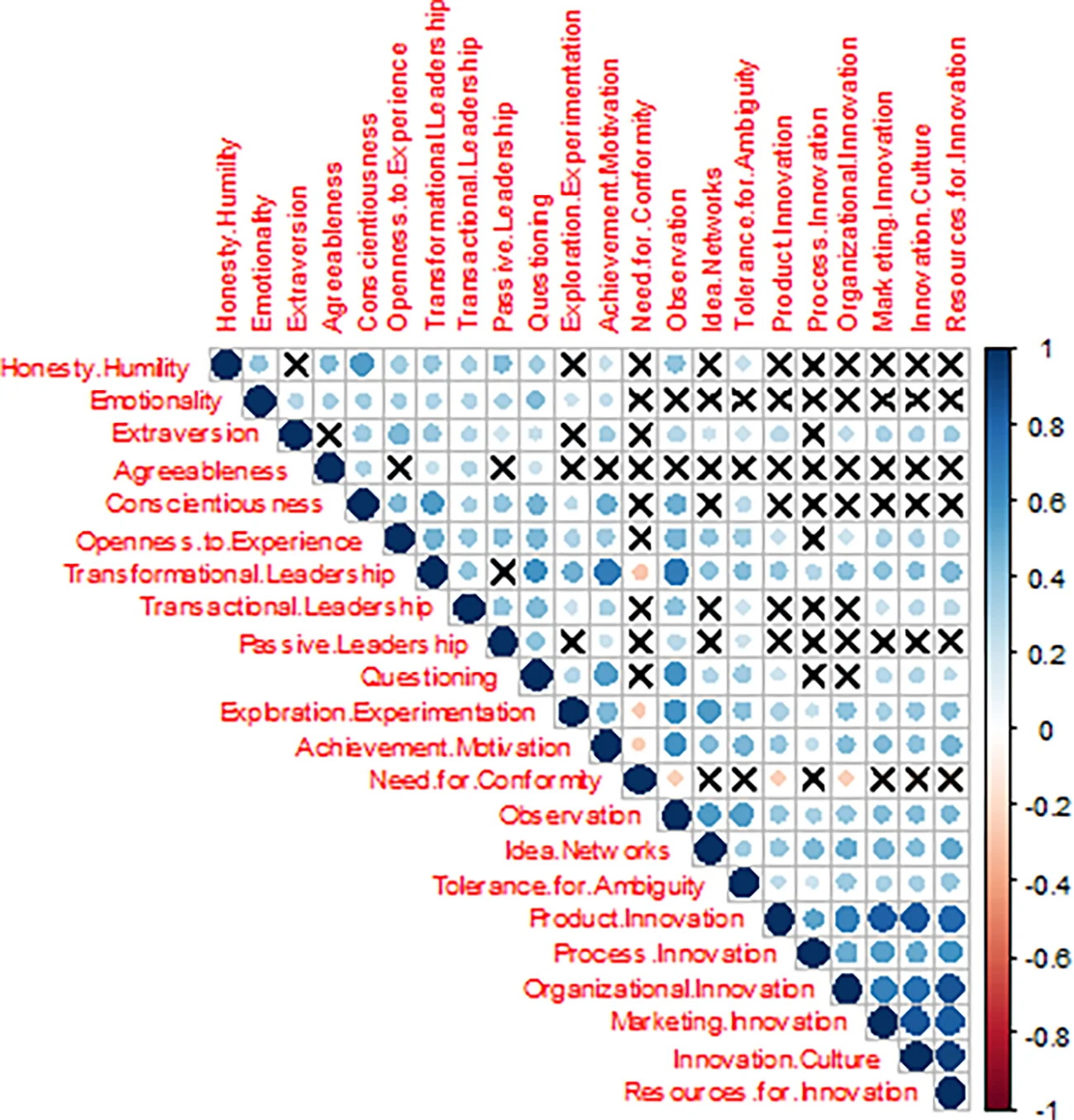
Correlation structure.

Figure 2 shows the pair-wise (Pearson) correlations. The correlation structure illustration uses a blue-white-red diverging color scale, where positive correlations are represented by blue marbles and negative correlations by red marbles. The intensity of the color and the marble size are both proportional to the correlation values. Non-significant correlations are marked with an “X.”

Preliminary results reveal significant positive correlations between openness to experience and transformational leadership (r = .46, p < .05), supporting Hypothesis 1. Additionally, transformational leadership showed strong, significant correlations with innovative behavior dimensions (all of them positive, except for Need for Conformity, which is r = -0.26), consistent with Hypothesis 2. Overall, innovative behavior dimensions also correlated positively with perceived innovation capabilities dimensions, with the following exceptions: i) the Questioning dimension exhibited positive, yet non-significant, correlations with Process Innovation and Organizational Innovation, ii) the Need for Conformity dimension exhibited negative correlations with all of the Innovation Capabilities dimensions, among which significance was reached only by the Need for Conformity – Product Innovation and Need for Conformity – Organizational Innovation pair-wise correlations. These outcomes partially (yet strongly) support Hypothesis 3.

These associations provide preliminary empirical support for the proposed model and suggest meaningful pathways through which CEO traits and leadership style may influence innovation outcomes in MSMEs. Further mediation analysis is presented in the next section to explore indirect effects.

### 3.3 Confirmatory path analysis

**Figure 3.**
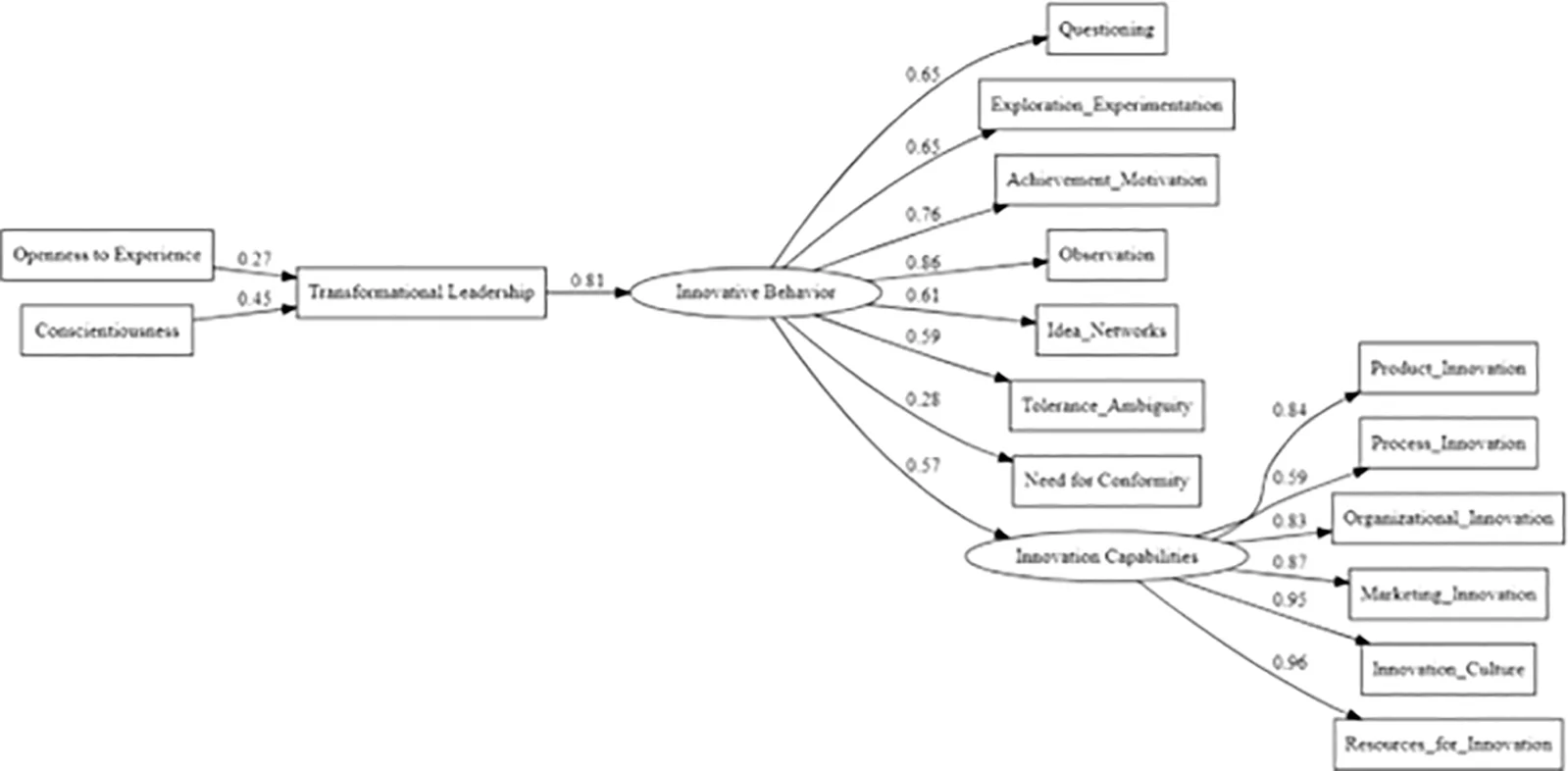
SEM model structure.

To examine the hypothesized relationships, we specified a confirmatory path analysis model in which Openness to Experience and Conscientiousness predicted Transformational Leadership, which in turn predicted Innovative Behavior. Innovative Behavior was modeled as a latent construct indicated by its seven subscales, and subsequently predicted Innovation Capabilities, modeled as a latent construct indicated by its six dimensions. This SEM model is illustrated in Figure 3.

Although HEXACO comprises six dimensions, prior research highlights Openness to Experience and Conscientiousness as the strongest predictors of leadership and innovation, and so does our initial exploratory analysis (as these show the strongest Cronbach’s Alpha values and consistency). Our SEM analysis confirmed that a model including only these two predictors achieved good fit indices, while adding all six traits yielded only marginal changes in fit but reduced model parsimony. We therefore retained the theory-driven, two-trait model as our primary analysis.

The model showed acceptable fit to the data, χ ^2^ = 195.29 (df = 102), p < .001; CFI = 0.92; TLI = 0.90; RMSEA = 0.09 (90% CI [.07, .11]); SRMR = 0.07. All hypothesized paths were statistically significant and in the expected direction. Specifically, both Openness to Experience (β = .27, p < .001) and Conscientiousness (β = .45, p < .001) positively predicted Transformational Leadership. Transformational Leadership in turn strongly predicted Innovative Behavior (β = .81, p < .001), which subsequently predicted Innovation Capabilities (β = .57, p = .001). Bootstrapped indirect effects further confirmed the mediating mechanisms: the effects of Openness to Experience and Conscientiousness on Innovative Behavior, as well as their serial effects on Innovation Capabilities through Transformational Leadership and Innovative Behavior, were significant.

Taken together, these results provide confirmatory evidence for the hypothesized mediation pathways linking CEO personality traits, leadership style, innovative behavior, and innovation capabilities.

Overall, the confirmatory path analysis confirms the theoretical model’s validity and emphasizes the role of transformational leadership as a conduit for converting individual traits into firm-level capabilities. These results reinforce the need to view innovation not merely as a function of strategy or resources, but also as an outcome of complex psychological and behavioral dynamics within top leadership.

## 4. Discussion

The findings of this study confirm the critical role of CEO personality and leadership style in shaping innovation outcomes in MSMEs operating in resource-constrained environments. The results offer preliminary empirical support for a path model where personality traits, particularly Openness to Experience, influence transformational leadership, which in turn enhances innovation-related behaviors and ultimately firm-level innovation capabilities.

These findings resonate with earlier research highlighting the mediating role of leadership in translating executive traits into strategic outcomes ( Mai, Do, & Phan, 2022). They also align with the work of ( Zettler et al., 2020), who identified strong associations between specific personality factors and leadership effectiveness. In particular, openness and conscientiousness appear to serve as psychological foundations that predispose leaders to adopt a transformational approach—characterized by vision, intellectual stimulation, and individualized consideration.

Moreover, our model reflects the sequential mediation logic explored by ( Obeidat et al., 2021), where behavioral mechanisms such as innovative behavior act as essential conduits between personality and performance outcomes. This view is reinforced by studies like that of ( Mismetti, Rovelli, Bettinelli, & Bergamaschi, 2025), who argue that innovation emerges not only from strategic intent but also from the CEO’s behavioral engagement with uncertainty and change. From a contextual perspective, our findings contribute to the limited empirical literature on MSMEs in Latin America. Unlike large corporations in developed markets, MSMEs in Colombia face persistent structural limitations. Here, the proactivity and behavioral orientation of CEOs toward innovation become crucial drivers of adaptation and competitive advantage.

Interestingly, this study also reinforces the idea that innovation is not a linear consequence of personality traits alone but the outcome of an integrated process where traits shape leadership styles, which in turn enable behavioral patterns aligned with innovation ( Bass & Avolio, 1996; Janssen, 2000). As noted by ( Geerts, 2024), understanding these multidimensional interactions provides a richer framework for designing leadership development interventions tailored to innovation.

Future research should explore the role of moderators such as CEO tenure ( Darouichi et al., 2021), organizational culture, and environmental turbulence to better understand the boundary conditions of the observed relationships. Longitudinal designs with larger and more diverse samples could also validate the temporal dynamics of the proposed mediation model.

In practical terms, the study suggests that innovation policies in emerging economies should move beyond firm-level subsidies or technology transfer schemes and invest in the psychological development of leadership within MSMEs. Training programs aimed at fostering transformational leadership and innovation-focused behaviors could amplify the latent potential of the sector.

Overall, this research contributes to advancing theoretical and practical understanding of how executive psychology intersects with leadership and behavior to drive innovation outcomes in constrained yet dynamic environments.

## 5. Conclusions

This study provides valuable initial evidence on how CEO personality traits, leadership styles, and innovative behaviors interact to shape innovation capabilities in Colombian MSMEs. By integrating the HEXACO personality framework, transformational leadership theory, and innovation behavior scales, the research advances a multidimensional perspective on the antecedents of innovation.

The findings indicate that CEOs with higher levels of openness to experience and conscientiousness are more likely to adopt a transformational leadership style, which in turn stimulates innovative behaviors and ultimately enhances firm-level innovation capabilities. This sequential relationship highlights the importance of considering both dispositional traits and behavioral processes in models of innovation leadership.

From a theoretical perspective, the study contributes to upper echelons research by demonstrating the value of integrated psychological-behavioral models in an emerging market context. From a practical standpoint, it underscores the need for leadership development programs tailored to MSMEs, focusing on fostering transformational leadership behaviors—such as intellectual stimulation, vision sharing, and individualized consideration—that translate personality traits into innovation outcomes. These findings can inform policymakers seeking to strengthen innovation ecosystems in emerging economies by highlighting the role of CEO psychology and leadership.

Despite its limitations, including a relatively small sample size, the study lays a foundation for future research using longitudinal designs and larger samples to validate the robustness of the proposed model. Further work could explore potential moderators such as CEO tenure, firm age, organizational culture, or environmental turbulence, as well as cross-national comparisons to deepen understanding of how executive psychology drives innovation trajectories in resource-constrained environments.

## Ethics and consent

This study was conducted in accordance with institutional ethical guidelines. Informed consent was obtained electronically from all participants prior to data collection. All personal and organizational data were de-identified, so that the CEOs’ identities and firms’ data are protected.

## Data Availability

All data underlying the results reported in this study are fully and openly available under the Creative Commons Attribution 4.0 International (CC BY 4.0) license. The datasets include all variables, descriptive statistics, and raw values used to compute means, standard deviations, and figures reported in the paper, as well as the data required to replicate the structural equation modeling (SEM) and correlation analyses. No embargo applies. All datasets are openly accessible and include the values used to compute reported statistics, generate figures, and reproduce all analyses. No data were excluded for privacy, ethical, or security reasons.
• Source code available from: https://github.com/iportnoy1/CEO-Dimensions-and-Their-Impact-on-Innovation-Capabilities-An-Empirical-Study-of-Colombian-MSMEs - • Archived data and materials available from (DOI): https://zenodo.org/records/17609131. • License: under an MIT and a CC BY 4.0 License, respectively.
