# Supplementary material for: CEO Dimensions and Their Mediating Role on Innovation Capabilities: An Empirical Study of Colombian MSMEs
Source: F1000Res. 2026 Jan 28;14:1313. Originally published 2025 Nov 26. [Version 2] doi: 10.12688/f1000research.171456.2 (PMC13560000; doi:10.12688/f1000research.171456.2)
Supplement: Peer review history

# Reviewer report: Reviewer response for version 2

**DOI:** 10.5256/f1000research.195512.r486849

**Published:** 2026-08-04

**Authors:** Cristian Guzmán Cofre

**Affiliations:** 1 Catholic University of the Most Holy Conception, Concepción, Chile

This paper explores an interesting topic in the field of innovation, as it analyzes how CEO characteristics influence companies’ innovation capabilities. This topic is relevant because it bridges the theory of corporate innovation—a well-established field in the literature—with issues of organizational innovation by including CEOs. This is particularly interesting because organizational innovation is a key determinant for medium-sized companies that seek to grow over time and improve their performance through innovation. This is because the literature recognizes that while small firms tend to innovate in products, large firms must innovate in processes; as a result, they may have lower economic performance compared to firms that do not innovate. 

 On the other hand, a strength of this study is its collection of data on CEO characteristics, since this information is not traditionally available in innovation surveys and is often correlated with managers’ educational background—though it does not accurately predict CEOs’ abilities to facilitate internal innovation processes. 

 ​​​​​​Furthermore, it is important to consider the characteristics of CEOs and the relevance of this study, since today smaller firms located in Latin America face more constraints or obstacles when it comes to spending on research and development activities, but given that knowledge is a public good, acquiring innovation skills through CEOs enables these types of companies to lower the barriers to implementing innovations and to efficiently utilize their internal innovation capabilities in alignment with the CEO’s leadership, personality, and skills.

Is the work clearly and accurately presented and does it cite the current literature?

Yes

If applicable, is the statistical analysis and its interpretation appropriate?

Yes

Are all the source data underlying the results available to ensure full reproducibility?

Yes

Is the study design appropriate and is the work technically sound?

Yes

Are the conclusions drawn adequately supported by the results?

Yes

Are sufficient details of methods and analysis provided to allow replication by others?

Yes

Reviewer Expertise:

innovación, estrategias de innovación, gasto en actividades de i+d.

I confirm that I have read this submission and believe that I have an appropriate level of expertise to confirm that it is of an acceptable scientific standard.

# Reviewer report: Reviewer response for version 2

**DOI:** 10.5256/f1000research.195512.r454001

**Published:** 2026-02-16

**Authors:** Ni Wayan Rustiarini

**Affiliations:** 1 Universitas Mahasaraswati Denpasar, Denpasar, Bali, Indonesia

This article has an unsystematic writing style. The ideal order is introduction, literature review, hypothesis, method, results, discussion, and conclusion. The theoretical model and hypothesis section should be written before the methods section and may be presented in a separate subsection (Literature Review and Hypothesis).

**Introduction**

 This article has a coherent narrative. However, the research gap remains normative rather than analytical. The author states a "lack of integrative models" but does not explicitly state the research gap. The author needs to address conceptual/empirical/methodological gaps and identify contradictions between previous findings. Furthermore, the research objectives are not explicitly formulated. The author also needs to include research contributions (both theoretical and practical).

**Method**

 The theoretical explanation should be presented in the theoretical model and hypothesis subsection. Sampling bias is very high: the population is large, but the sample size is only 106 CEOs. This number is statistically too small for generalizing to the population. Selecting a sample using convenience can also lead to a typical elite response. There are issues with the pilot test; it states that a subsample of 106 CEOs was piloted. This means the number of pilot tests equals the final sample size. This means the author did not conduct a specific pilot test; the pilot test was the same as the main study. The variable measurement indicators need to be explicitly stated. All variables were measured using a self-reported CEO survey, which required a common-methods bias test (Harman single-factor test). The author also did not explain the measurement model, nor did he report factor loadings, AVE, discriminant validity, or HTMT ratio.

**Theoretical Model and Hypothesis**

 The author needs to formulate hypotheses based on the theory underlying this research. Each hypothesis has its own explanation, supported by the findings of previous studies. Figure 1 is difficult to understand; it needs to be explained, including the direct and indirect relationships.

**Results**

 The discussion only confirms the hypothesis; there are no unexpected findings, theoretical contradictions, or alternative explanations. The discussion tends to state that the results align with prior research. The author also needs to include a robustness analysis section.

**Conclusion**

 The author claims to advance upper-echelon research, but the model only tests existing relationships (does not introduce new constructs or change theory). Therefore, this is not a theoretical advancement, but rather an empirical replication. The limitations section is unclear, as it mentions only the small sample size. However, the main limitations are common method bias, sampling bias, and measurement issues.

Is the work clearly and accurately presented and does it cite the current literature?

Partly

If applicable, is the statistical analysis and its interpretation appropriate?

Partly

Are all the source data underlying the results available to ensure full reproducibility?

No

Is the study design appropriate and is the work technically sound?

No

Are the conclusions drawn adequately supported by the results?

No

Are sufficient details of methods and analysis provided to allow replication by others?

Partly

Reviewer Expertise:

Economic

I confirm that I have read this submission and believe that I have an appropriate level of expertise to state that I do not consider it to be of an acceptable scientific standard, for reasons outlined above.

# Reviewer report: Reviewer response for version 2

**DOI:** 10.5256/f1000research.195512.r453493

**Published:** 2026-02-09

**Authors:** Diogo Ferraz

**Affiliations:** 1 University of Sao Paulo, São Paulo, State of São Paulo, Brazil

All my questions are adequately answered.

Is the work clearly and accurately presented and does it cite the current literature?

Yes

If applicable, is the statistical analysis and its interpretation appropriate?

Yes

Are all the source data underlying the results available to ensure full reproducibility?

Yes

Is the study design appropriate and is the work technically sound?

Yes

Are the conclusions drawn adequately supported by the results?

Yes

Are sufficient details of methods and analysis provided to allow replication by others?

Yes

Reviewer Expertise:

Economic Complexity, Innovation, Sustainable Development.

I confirm that I have read this submission and believe that I have an appropriate level of expertise to confirm that it is of an acceptable scientific standard.

# Reviewer report: Reviewer response for version 1

**DOI:** 10.5256/f1000research.189066.r443221

**Published:** 2026-01-12

**Authors:** Hatinah Abu Bakar

**Affiliations:** 1 Universiti Teknologi Mara, Shah Alam, Malaysia

**Topic:** 

 The title "CEO Dimensions and Their Impact on Innovation Capabilities: An Empirical Study of Colombian MSMEs" is straightforward and informative; advisable, it is recommended to include "mediation" as a major contribution of the article lies in testing a mediation model.

**Abstract**

 The abstract well written nonetheless, it does not clearly address the mediating function of essential constructions within the suggested model.

**Introduction** 

 This study focuses on MSMEs in Colombia, however there is a lacking of information about Colombian MSMEs. Moreover the problem statement does not adequately address the challenges  or issues and research gaps related to Colombian MSMEs.

 The paragraph is well written and conceptually coherent; however, several cited sources exceed the commonly preferred 5-year. 

 Moreover, there is lacking discussion on transformational leadership as mediator.

**Methods**

 The paragraph clearly states the target population (CEOs or top executives of MSMEs in Colombia) and appropriately justifies the use of purposive and convenience sampling given practical constraints. However, to strengthen the sampling justification and enhance the generalizability of the study, the authors are encouraged to provide official statistics on the total number of MSMEs in Colombia.

 It is advisable for the authors to provide **more detailed information regarding the screening and inclusion criteria**, including the procedures used to identify and select eligible CEOs for the study

 The description of SEM could be more precise by indicating whether CB-SEM or PLS-SEM was employed and why this approach was appropriate for the study’s objectives (e.g., theory testing vs. prediction).

**Results**

 Advisable do not used word "WE". Can change "to this study" or "this research".

 Advisable for correlational analysis explained in result not as a correction structure (figure 2) 

**Discussion and Conclusion**

 Well discussed.  

**Overall Comment. **: 

 The authors are encouraged to review the overall flow and organization of the manuscript. In particular:

 (i) The placement of Section 2.6 (Ethical Considerations) may be reconsidered, as its current location may not best align with the manuscript structure.

 (ii) The literature discussion could be further refined to ensure clearer linkage with the research objectives.

 (iii) The statistical analysis and results sections may be combined into a single **Findings and Analysis** section to enhance clarity and coherence.

Is the work clearly and accurately presented and does it cite the current literature?

Partly

If applicable, is the statistical analysis and its interpretation appropriate?

Partly

Are all the source data underlying the results available to ensure full reproducibility?

Partly

Is the study design appropriate and is the work technically sound?

Partly

Are the conclusions drawn adequately supported by the results?

Yes

Are sufficient details of methods and analysis provided to allow replication by others?

Partly

Reviewer Expertise:

My research interests encompass entrepreneurship, management, and organizational behavior, particularly in relation to organizational strategy, leadership, and entrepreneurial performance.

I confirm that I have read this submission and believe that I have an appropriate level of expertise to confirm that it is of an acceptable scientific standard, however I have significant reservations, as outlined above.

# Response

**Published:** 2026-01-20

**Authors:** Tobias A. Parodi-Camano

**Affiliations:** Industrial Engineering, Universidad de Cordoba, Montería, Cordoba, Colombia

**Reviewers’ Remark:**

 1. The title "CEO Dimensions and Their Impact on Innovation Capabilities: An Empirical Study of Colombian MSMEs" is straightforward and informative; advisable, it is recommended to include "mediation" as a major contribution of the article lies in testing a mediation model.

**Change or Comment:**

 We have modified the title to stress the use of mediation in the model. The updated title is: “CEO Dimensions and Their Mediating Role on Innovation Capabilities: An Empirical Study of Colombian MSMEs”

**Reviewers’ Remark:**

 2. The abstract well written nonetheless, it does not clearly address the mediating function of essential constructions within the suggested model.​​​​​​​

**Change or Comment:**

 We have modified the abstract to make highlight mediation more explicitly.​​​​​​​

**Reviewers’ Remark:**

 3. **Introduction**

 This study focuses on MSMEs in Colombia, however there is a lacking of information about Colombian MSMEs. Moreover the problem statement does not adequately address the challenges  or issues and research gaps related to Colombian MSMEs.

 The paragraph is well written and conceptually coherent; however, several cited sources exceed the commonly preferred 5-year.

 Moreover, there is lacking discussion on transformational leadership as mediator.

**Change or Comment:**

 We have strengthened the Introduction section by explicitly discussing the structural and innovation-related challenges faced by Colombian MSMEs, including limited R&D investment, institutional constraints, and weak innovation ecosystems. Also, we have clarified how these contextual factors amplify the role of CEOs in fostering innovation outcomes, thus reinforcing the relevance of our research gap in the Colombian MSMEs context.

 Additionally, we have included more recent (from 2021 onward) to support the research gap identification and give support to the research hypotheses. Nevertheless, we consider that some older research works provide a strong theoretical background, as well as some others are seminal works that are relevant to this date. Thus, we have kept some of them.

**Reviewers’ Remark:**

 4. **Methods**

 The paragraph clearly states the target population (CEOs or top executives of MSMEs in Colombia) and appropriately justifies the use of purposive and convenience sampling given practical constraints. However, to strengthen the sampling justification and enhance the generalizability of the study, the authors are encouraged to provide official statistics on the total number of MSMEs in Colombia.

 It is advisable for the authors to provide more detailed information regarding the screening and inclusion criteria, including the procedures used to identify and select eligible CEOs for the study

 The description of SEM could be more precise by indicating whether CB-SEM or PLS-SEM was employed and why this approach was appropriate for the study’s objectives (e.g., theory testing vs. prediction).

**Change or Comment:**

 To strengthen the sampling justification and contextualize the scope of the target population, we have added (in Subsection 2.2 - Participants and Sampling) official statistics indicating that Colombia has more than 1.7 million MSMEs, representing over 90% of the country’s productive units. This information highlights the scale of the population under study and further supports the use of purposive and convenience sampling given the practical constraints associated with accessing CEOs of MSMEs.

 Moreover, we have added the inclusion criteria used for the CEOs screening process (in Subsection 2.2 - Participants and Sampling).

 We have clarified in the Methods section (in Subsection 2.5) that a covariance-based SEM (CB-SEM) approach was employed using the lavaan package in R. We further justified this choice by noting that the study is theory-driven and focused on hypothesis testing and mediation analysis rather than prediction

**Reviewers’ Remark:**

 5. **Results**

 Advisable do not used word "WE". Can change "to this study" or "this research".

 Advisable for correlational analysis explained in result not as a correction structure (figure 2)​​​​​​​

**Change or Comment:**

 We have changed the pronoun “we” for “this study” (or something along those lines) as suggested throughout the entire manuscript.

**Reviewers’ Remark:**

 6. **Discussion and Conclusion**

 Well discussed. 

**Change or Comment:**

 We thank the reviewer for their feedback on this matter.

**Reviewers’ Remark:**

 7. **Overall Comment**

 The authors are encouraged to review the overall flow and organization of the manuscript. In particular:

 (i) The placement of Section 2.6 (Ethical Considerations) may be reconsidered, as its current location may not best align with the manuscript structure.

 (ii) The literature discussion could be further refined to ensure clearer linkage with the research objectives.

 (iii) The statistical analysis and results sections may be combined into a single Findings and Analysis section to enhance clarity and coherence.

**Change or Comment:**

**Overall Comment**

 The authors are encouraged to review the overall flow and organization of the manuscript. In particular:

 (i) The placement of Section 2.6 (Ethical Considerations) may be reconsidered, as its current location may not best align with the manuscript structure.

 (ii) The literature discussion could be further refined to ensure clearer linkage with the research objectives.

 (iii) The statistical analysis and results sections may be combined into a single Findings and Analysis section to enhance clarity and coherence.

# Reviewer report: Reviewer response for version 1

**DOI:** 10.5256/f1000research.189066.r437424

**Published:** 2026-01-07

**Authors:** Diogo Ferraz

**Affiliations:** 1 University of Sao Paulo, São Paulo, State of São Paulo, Brazil

The article "CEO Dimensions and Their Impact on Innovation Capabilities" offers a relevant approach for Latin American countries, employs an appropriate method, and presents interesting findings that other researchers in developing regions might replicate. Some revisions must be made before acceptance.

 1. Please, improve the storyline of your paper. The Introduction must be reviewed to present a relevant research gap.

 2. Please, provide a paragraph with all the contributions of this paper and present citations to prove each contribution. 

 3. Please provide better policy recommendations or practical implications. The current version is weak.

 4. Please review the entire Conclusions section. Rewrite more critically.

Is the work clearly and accurately presented and does it cite the current literature?

Yes

If applicable, is the statistical analysis and its interpretation appropriate?

Yes

Are all the source data underlying the results available to ensure full reproducibility?

Yes

Is the study design appropriate and is the work technically sound?

Yes

Are the conclusions drawn adequately supported by the results?

Yes

Are sufficient details of methods and analysis provided to allow replication by others?

Yes

Reviewer Expertise:

Economic Complexity, Innovation, Sustainable Development.

I confirm that I have read this submission and believe that I have an appropriate level of expertise to confirm that it is of an acceptable scientific standard, however I have significant reservations, as outlined above.

# Response

**Published:** 2026-01-20

**Authors:** Tobias A. Parodi-Camano

**Affiliations:** Industrial Engineering, Universidad de Cordoba, Montería, Cordoba, Colombia

**Reviewers’ Remark:**

 1. Please, improve the storyline of your paper. The Introduction must be reviewed to present a relevant research gap.

**Change or Comment:**

 We have revised the Introduction to strengthen the narrative progression from prior literature to the study’s objectives. Specifically, we clarified how existing research on CEO personality, leadership, and innovation remains scarce, particularly in the context of MSMEs in emerging economies. Then, we stressed how the particular conditions faced by Colombian MSMEs highlights the relevance of CEO-level factors, making the motivation for the proposed integrative model clear. Also, we updated the literature base with recent papers (from 2021 onward) to support the gap identification and hypotheses, while retaining seminal works that provide essential theoretical foundations.

**Reviewers’ Remark:**

 2. Please, provide a paragraph with all the contributions of this paper and present citations to prove each contribution.

**Change or Comment:**

 We have included a paragraph in the Conclusions section outlining the main contributions of the study. Each contribution is backed by relevant prior research works.

**Reviewers’ Remark:**

 3. Please provide better policy recommendations or practical implications. The current version is weak.

**Change or Comment:**

 We have added two paragraphs within the Discussion section providing more detailed and comprehensive policy recommendations and implications stemmed from the main findings of our research. 

**Reviewers’ Remark:**

 4. Please review the entire Conclusions section. Rewrite more critically.

**Change or Comment:**

 We have carefully rewritten the Conclusions to adopt a more critical perspective. Now it synthesizes the main findings, explicitly distinguishes between theoretical and practical contributions, acknowledges the key limitations of the study, and outlines concrete directions for future research. Moreover, we consolidated the discussion of contributions to emphasize the study’s added value in relation to existing leadership and innovation literature.
